# Identification and functional analysis of five genes that encode distinct isoforms of protein phosphatase 1 in *Nilaparvata lugens*

**DOI:** 10.1038/s41598-020-67735-7

**Published:** 2020-07-02

**Authors:** Weixia Wang, Tingheng Zhu, Fengxiang Lai, Pinjun Wan, Qi Wei, Qiang Fu

**Affiliations:** 1 State Key Lab of Rice Biology, China National Rice Research Institute, Hangzhou, 310006 Zhejiang China; 2 College of Biotechnology and Biongineering, Zhejiang University of Technology, Chaowang Road, Hangzhou, 310014 Zhejiang China

**Keywords:** Biotechnology, Genetics, Molecular biology

## Abstract

Ten distinct cDNAs encoding five different protein phosphatases 1 (PPP1) were cloned from *Nilaparvata lugens*. *NlPPP1α* and *NlPPP1β* are highly conserved whereas *NlPPP1-Y*, *NlPPP1-Y1* and *NlPPP1-Y2* are lowly conserved among insects. NlPPP1α and NlPPP1β exhibited a ubiquitous expression, while *NlPPP1-Y*, *NlPPP1-Y1*, and *NlPPP1-Y2* were obviously detected from the 4th instar nymph to imago developmental stages in males, especially detected in internal reproductive organ and fat bodies of the male. Injection nymphs with dsRNA of *NlPPP1α* or *NlPPP1β* was able to reduce the target gene expression in a range of 71.5–91.0%, inducing a maximum mortality rate of 95.2% or 97.2% at 10th day after injection and eclosion ratio down by 65.5–100.0%. Injection with dsNlPPP1Ys targeted to *NlPPP1-Y*, *NlPPP1-Y1*and *NlPPP1-Y2* was able to induce a maximum mortality rate of 95.5% at 10th day after injection, eclosion ratio down by 86.4%. Knock-down one of the male-biased *NlPPP1* genes has no effect on survival and eclosion ratio. Injection of 4th instar nymph with dsNlPPP1Ys led to reduced oviposition amount and hatchability, down by 44.7% and 19.6% respectively. Knock-down of *NlPPP1-Y1* or *NlPPP1-Y2* gene did not significantly affect oviposition amount but significantly affected hatchability. The results indicate that the male-biased *NlPPP1* genes have overlapping functions in *N. lugens* development, and *NlPPP1-Y1* and *NlPPP1-Y2* may play important roles in spermatogenesis and fertilization. The dsNlPPP1β and dsNlPPP1Ys in this study could be the preferred sequence in RNAi and low-conserved male-biased *NlPPP1* genes could be potential target for *N. lugens* control.

## Introduction

The Brown planthopper (*Nilaparvata lugens,* Hemiptera: Delphacidae) is one of the most destructive rice pest, which causes serious damage to rice crop through phloem sap sucking and nutrient depletion^1^. In the last decades, the main means of pest control for *N. lugen*s is to apply pesticides and resistant rice varieties. However, *N. lugens* has developed resistance against most of the insecticides and adaptation to resistant rice varieties^2,3^. With the development of molecular biology of pests, cloning and identifying the genes related to the growth and development of pests can help us to understand the virulent mechanism of pests, and also bring chances for finding new molecular targets and designing alternative control strategies.

Protein phosphorylation is a common means to the regulation of most cellular processes^4^. This is highlighted by the fact that protein kinases and phosphatases, which respectively add and remove phosphate on proteins, constitute 2–4% of the genes in a typical eukaryotic genome^5^. Protein phosphatases (PPP) can be classified into three groups: serine/threonine phosphatases, the protein Tyr phosphatase superfamily, and Asp-based protein phosphatases. In eukaryotic cells, eight types of serine/threonine phosphatases have been identified^6^. Among these, PPP1 is a ubiquitously expressed, highly conserved and abundant eukaryotic protein serine/threonine phosphatase that regulates diverse cellular processes such as cell-cycle progression, protein synthesis, muscle contraction, glycogen metabolism, transcription and neuronal signalling^7–9^. Each functional PPP1 enzyme consists of a catalytic subunit and a regulatory subunit. Catalytic subunit has three subunits-PPP1α, PPP1β and PPP1γ^10^, and can form as many as 650 distinct complexes with PPP1-interacting proteins^11^. It is estimated that around one third of all eukaryotic proteins are dephosphorylated by PPP1. The catalytic subunit of PPP1 is highly conserved among all eukaryotes, with approximately 70% or greater protein sequence identity in any pairwise alignment^12^. In *Drosophila Melanogaster*, PP1α isozyme encoded by three genes named after their respective chromosomal location: PP1α13C (FlyBase: Pp1-13C), PP1α87B (Pp1-87B), and PP1α96A (Pp1α-96A). Only one gene, PP1β9C (flapwing, flw), encodes the PP1β type^13,14^, and PpY-55A, PpN-56A, PpD5, PpD6, Pp1-Y1 and Pp1-Y2, which are Drosophila specific intron-less phosphatases with male biased expression^15^. In Drosophila, PPP1α is essential for mitosis^13,16,17^. Loss of PPP1β leads to increased levels of actin disorganization, crumpled or blistered wings^18,19^ and disrupt oocyte polarization^20^. PPP1 was suggested to be involved in the regulation of glycolysis which plays important roles in the internal metabolism of *Spodoptera litura* during metamorphosis^21^. PPP1 in tick play a role in modulating tick salivary secretion^22^. In fungal pathogen *Candida albicans*, PPP1 may contribute to pathogenicity^23^. For male biased PPP1, deletion of the testis-specific PPP1γ2 gene in mice results in defective sperm development and motility^24–26^. Also in nematode *Caenorhabditis elegans*, sperm specific PPP1 phosphatases are required for chromosome segregation during sperm meiosis and necessary for the ability of sperm to fertilize^27^. Armstrong et al. reported that a male specific protein phosphatase PPY in Drosophila may be required to prevent cyst cell division, increase transcription for provision of nutrients to the germ cells and/or provide a signal for spermatocyte differentiation^28^.

In summary, PPP1 is an important functional gene for eukaryotic growth and development and metabolic regulation. However, the PPP1 family genes in *N. lugens* has not been revealed, and whether they can be used as targets for controlling *N. lugens* has not been explored. In this study, we report the isolation of ten cDNA clones encoding five distinct catalytic subunits of type 1 protein phosphatases *(NlPPP1α*, *NlPPP1β*, *NlPPP1-Y*, *NlPPP1-Y1* and *NlPPP1-Y2*) from *N. lugens*. To explore a potential role for PPP1 in *N. lugens*, the present study examined the mRNA expression levels of the *NlPPP1* during the nymph development and in diverse tissue. The effects of knockdown the expression of *NlPPP1* by RNAi method were also examined. These results demonstrated that *NlPPP1α, NlPPP1β* and *NlPPP1-Y* play important roles in *N. lugens* development. Male biased *NlPPP1-Y1* and *NlPPP1-Y2* play important roles in spermatogenesis and fertilization ability. Our data also reveals *NlPPP1Ys* can be the preferred targets for *N. lugens* control by means of RNAi.

## Materials and methods

### Insects and sampling

*Nilaparvata lugens* were collected in field of China National Rice Research Institute, Fuyang, Zhejiang, China and reared on the susceptible rice variety Taichung Native1 in wire mesh cages at 27 ± 2 °C with 80 ± 5% relative humidity under a 16 h light/8 h darkness photoperiod.

Adult females and males, 2 days after eclosion, were immobilized by placing them in a freezer for 15 min, and their midguts (50), salivary glands (100), fat bodies (50) and internal reproductive organs (50) were dissected with tweezers. Eggs (200) and the individuals from the day 1 of the 1st instar (100), 2nd (100), 3rd (50), 4th (20) to day 3 of the 5th instar nymphs (10) and newly emerged female (10) and male adults (10) were randomly selected respectively. The number of insects in each sample is given in parentheses above. All samples were collected in triplicate. The samples were frozen in liquid nitrogen and stored at − 80 °C prior to RNA extraction.

### RNA isolation, sequence amplification and analysis

Total RNA was isolated from *N. lugens* at different developmental stages and from different tissues using an RNeasy Mini Kit (Qiagen, Hilden, Germany) according to the manufacturer’s instructions. The RNA was quantified and the quality verified by NanoDrop 2000 spectrophotometer (Thermo Fischer Scientific, Bremen, Germany). A total of 500 ng RNA was used for reverse transcription in a 10 μL reaction with the ReverTra Ace qPCR RT Master Mix with gDNA Remover Kit (ToYoBo, Osaka, Japan). Synthesized cDNA was diluted tenfold and used as template for quantitative PCR.

The PPP1 gene was amplified with primer pairs which based on our transcriptome database from whole bodies of *N. lugens* and designed using National Center for Biotechnology Information (NCBI) primer design tool (www.ncbi.nlm. nih.gov/tools/primer-blast). All the primer used in this study were synthesized by Invitrogen Co., Ltd Shanghai China and listed in Table 1. The polymerase chain reaction (PCR) procedure was as follows: 94 °C for 5 min followed by 40 cycles of 94 °C for 30 s, 58 °C for 45 s, and 72 °C for 60 s. The samples were then incubated for 10 min at 72 °C. The PCR products were gel-purified and cloned into the PCR^2.1^Topo vector (Invitrogen, China) and then the plasmids from positive colonies were sequenced with the M13 primer pair on ABI Prism 3100 DNA sequencer (Invitrogen Co., Ltd Shanghai China).The cDNA sequences were analysis with BLAST against *N. lugens* genome (*Nilaparvata lugens* (taxid: 108931)).The open reading frame (ORF) was determined using ORF Finder (https://www.ncbi.nlm.nih.gov/gorf/gorf.html). The translated amino acid sequence was used as a query to identify homologous proteins and compared with other PPP1 deposited in GenBank using the BLASTp tool (https://blast.ncbi.nlm.nih.gov/Blast.cgi). The molecular weight (Mw) and isoelectric point (pI) of NlPPP1 were calculated by the Compute pI/Mw tool (https://web.expasy.org/compute_pi/). The phylogenetic tree of PPP1 was constructed using the maximum likelihood method with MEGA 5.0(https://megasoftware.net/). All the PPP1 sequences from *N. lugens* were aligned in a multiple sequences alignment using CLUSTAL X and edited with GeneDoc software. The phosphorylation sites were predicted using Netphos3.0 server (https://www.cbs.dtu.dk).

**Table 1:** Primers used in this study.

Primers	Forward primer sequence	Reverse primer sequence
**For clone**		
NlPPP1α	CTCACTTCGTTCGTTCGCATT	AGACCTACTCCAGGTAGCCTT
NlPPP1β	CGTAGACGTCGGTCTGTGTG	ACCTAATGCTTTGCTGTTCCTT
NlPPP1-Y	ATTTACGCGGGTCTTGTGAAC	CGTTCTCGGTCCTTCTTCCTCTT
NlPPP1-Y1	GTTGTATGCTGGACAACACTG	GAAAAAGCATCTCTTAAACCCG
NlPPP1-Y2	TCAATCTTGAACCCTTTGTGTGAA	GCGCTTTTTATCTCGTTCCGC
**For RT-qPCR**		
qNlPPP1α	TCGGAAATCCGTGGATTGTGT	TCGGAAATCCGTGGATTGTGT
qNlPPP1β	AGGCATGATGTCTGTCGATGAA	ACAGCGACCTCCCTTTGAG
qNlPPP1-Y	ATGTAGCTGGGTTCCCTCCC	ACTCGTGGTTGCCTCGTACC
qNlPPP1-Y1	TGAGATGGTTTGACTCCGCC	TCGAGGCACACTCATGGTTC
qNlPPP1-Y2	CACCCCTAAAGGTGGTTGGAG	CCCACGCAACATGAAGAAGTT
**For dsRNA synthesis**		
dsNlPPP1α	TAATACGACTCACTATAGGG TTTCGAGTACGGAGGATTCCC	TAATACGACTCACTATAGGG GCATTGGCAACTTCTCTCCAC
dsNlPPP1β	TAATACGACTCACTATAGGG ACTCTGGTCGTCCATCTACGC	TAATACGACTCACTATAGGG TCATCAGGGTCCATAAATTGGGT
dsNlPPP1-Y	TAATACGACTCACTATAGGG ATTTACGCGGGTCTTGTGAAC	TAATACGACTCACTATAGGG ACTCAGACTCACCAGAGGGTT
dsNlPPP1-Y1	TAATACGACTCACTATAGGG GTTGTATGCTGGACAACACTG	TAATACGACTCACTATAGGG GCCCTTCACTTCTAGCAGCC
dsNlPPP1-Y2	TAATACGACTCACTATAGGG TGAACCCTTTGTGTGAATTTGAAGA	TAATACGACTCACTATAGGG AGCAGATCGCAGATCAACCC
dsNlPPP1Ys	TAATACGACTCACTATAGGG GCGGCTCAAAACAGTATCACAG	TAATACGACTCACTATAGGG GGCGAAGAACTCGTAGCCAT

### Expression analysis by real-time quantitative PCR (RT-qPCR)

*NlPPP1* transcript levels were quantified in tissues and development stages with specific primers for *NlPPP1α*, *NlPPP1β*, and *NlPPP1-Ys* which were designed based on the cDNA sequence obtained. The RT-qPCR (20 μL per reaction) used 3.0 μL cDNA template, 0.4 μL of each primer (10 mM) and 10 μL SYBR Premix (Toyobo, Japan). RT-qPCRs were carried out using an ABI 7,500 Real-time PCR system (Applied Biosystems, Carlsbad, CA, USA) in a two-step reaction (3 min denaturation at 95 °C, 40 cycles 10 s denaturation at 95 °C, 30 s annealing/extension at 60 °C) followed by a melt curve analysis at the end of the run. Each experiment consisted of 3 separate biological replicates, each of which was comprises 3 technical replicates. Relative expression levels were calculated using the 2^−∆∆CT^ method^29^. The *N. lugens* housekeeping genes for β-actin (FJ948574) and 18S rRNA (JN662398) were used as the reference genes^30^. Fold induction values of target genes were calculated with the ΔΔCt equation and normalized to the mRNA level of target genes in control which were defined as 1.0.

### RNAi interference and bioassay

The dsRNA synthesis, microinjection experiment and bioassay was conducted based on our previously described method^31^. A PCR method using the plasmid with *NlPPP1* insert as template was used to generate the templates for the dsRNA synthesis. The GFP gene (ACY56286) was used as a control. Approximately 70 ng of dsRNA was injected into each newly moulted third-instar nymphs or fourth-instar nymphs (3-day old) *N. lugens.* The survived nymphs in each treatment were selected and reared on 60- to70 day-old rice variety TN1 in one cage. A total of 175 nymphs (5 replicates, 35 individuals in each replicate) for each treatment were used for each dsRNA injection. RNAi efficiency for dsNlPPP1α and dsNlPPP1β was assessed at 4 days post injection by RT-qPCR. Total RNA was extracted from 5 nymphs sampled from each treatment and each replicate. RNAi efficiency for male-biased gene was assessed at 4 days after emergency by RT-qPCR, internal reproductive organs dissected from approximately 5 males were used in each sample.

The survival rates of the injected 3rd-instar nymphs were observed at 24 h intervals with duration of 10 days. Once the 4th-instar nymphs after injection emerged, each female was matched with one male and each pair was allowed to reproduce separately. For the male-biased gene, the injected male was matched with untreated female. In total, 15 single pairs per gene were successfully mated. The number of newly hatched nymphs was recorded every other day until no more nymphs were observed for two successive days. The number of unhatched eggs was also recoded under a light microscope. Eggs were scraped from the leaf sheaths and blades using a pin. All analyses were performed with the data procession system (DPS) of Tang and Feng^32^. Duncan’s tests were used to determine differences between the treatment and control. Values of P < 0.05 were considered significantly, all values were expressed as mean ± SEM.

### Enzyme-linked immune sorbent assay (ELISA) analysis

Two days after emergence, a total of 40 male internal reproductive organs (4 replicates, 10 individuals in each replicate) were randomly selected and dissected from the treatment group injected with dsNlPPP1Ys and the control group injected with dsGFP. A total of 20 female internal reproductive organs (4 replicates, 5 individuals in each replicate) were randomly selected and dissected from females mated with injected with dsNlPPP1Ys and females mated with injected with dsGFP. The male and female internal reproductive organs were homogenized with a glass tissue grinder in 200 μL 0.8% NaCl buffer. Homogenates were centrifuged at 10,000×*g* for 15 min at 4 °C, and the supernatants were used for total protein (TP) determination with the Insect TP Elisa Kit (Boshen Biotech Co ltd, Nanjing Jiangsu China). The optical density was read at 450 nm on a Sunrise ELISA reader (Tecan, Mannedorf, Switzerland).

## Results

### Sequence analysis of *NlPPP1*

**Figure 1:**
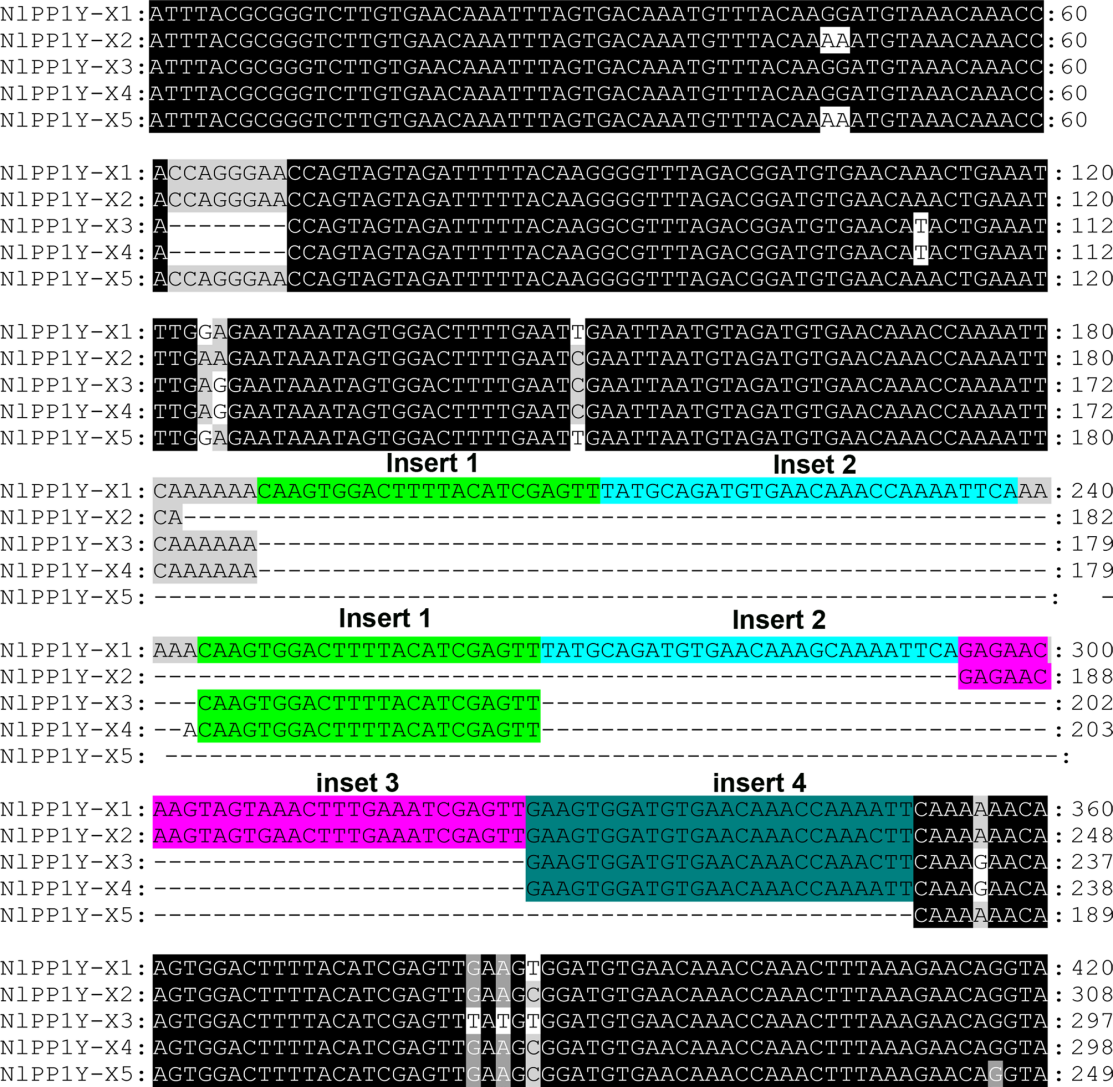
The 5′-UTRs alignment of six *NlPPP1-Y* transcript variants with CLUSTAL X. The insert sequences shaded in same color indicate the identical nucleic acids. Letters shaded in black and gray indicate the identical and similar nucleic acids, respectively.

Based on the assembled transcriptome which constructed in our laboratory, five different pair of PCR primers were designed and used to clone the PPP1 gene from *N. lugens*. Totally 10 cDNA clones were isolated. Based on the comparative study of their nucleotide and deduced amino acid sequences with those reported, these cDNA clones were named *NlPPP1α*, *NlPPP1β*, *NlPPP1-Y1*, *NlPPP1-Y2* and *NlPPP1-Y.* There are 6 transcript variants were identified for *NlPPP1-Y* named as *NlPPP1-Y-X1-6* (listed in Table 2). Sequence analysis showed the difference among the six transcripts was mainly caused by diverse inserts pattern (Fig. 1). The cDNA sequences were blasted against NCBI and *N. lugens* genomic data. Ten cDNA sequences have one or more blast hits with ≥ 90% query cover and E-value 0.0. Three blast hits (XM_022340879, XM_022340880 and XM_022340881) for *NlPPP1-Y* transcript variants were retained, which share common 5-UTR (582 bp in length) and ORF (1,005 bp in length) and three different lengths 3-UTR (233, 66 and 32 bp respectively). The 5-UTR is the same with *NlPPP1-Y-X6*.

**Table 2:** PPP1 genes cloned from *N. lugens* and their sequence characteristics.

Gene ID	Blastn NCBI Accession No. (length bp^1^)	Blast *N. lugens* genome	Accession no.	Exon no.	Full length bp^1^	ORF bp^1^	5′-UTR bp	3′-UTR bp	CDS aa	Mw kDa	pI
NlPPP1α	XM_022344876 (1,807)	Scaffold2314	MN031258	7	1,263	987	120	156	329	37.5	6.29
NlPPP1β	XM_022349475 (3,628)	Scaffold31341	MN031259	3	1,731	978	31	722	326	37.2	6.25
NlPPP1-Y-X1 NlPPP1-Y-X2 NlPPP1-Y-X3 NlPPP1-Y-X4 NlPPP1-Y-X5 NlPPP1-Y-X6	XM_02234088 (1,820) XM_02234088 (1,619) XM_02234087 (1,653)	Scaffold1294	MN647767 MN647768 MN647769 MN647770 MN647771 MN647772	4	1,627 1,515 1,503 1,501 1,456 1,447	1,005	599 487 475 473 428 421	23	335	37.9	5.48
NlPPP1-Y1	XM_02233082 (1,612)	Scaffold10	MN480456	1	1,415	936	465	14	312	35.6	5.85
NlPPP1-Y2	XM_02234221 (1,728) XM_02234910 (1,597)	Scaffold1537 Scaffold5497	MN480457	1	1,158	936	81	141	312	35.7	5.85

*bp* base pair, *aa* amino acid, *Mw* molecular weight, *pI* protein isoelectric point.

Analysis of the genomic position and structure showed that *NlPPP1α*, *NlPPP1β*, *NlPPP1-Y*, *NlPPP1-Y1* and *NlPPP1-Y2* contains 7, 3 and 4 exon and is located at scaffold 2,314, 31,341, 1,294, 10 and 1,537 in the *N. lugens* genome respectively. No intron was found in cDNA sequences of *NlPPP1-Y1* and *NlPPP1-Y2*. The blast information, deduced amino acid lengths, molecular weights and isoelectric points of the *NlPPP1* cDNA clones are illustrated in Table 2.

The percent identities of ORF *NlPPP1α* and *NlPPP1β* is 66.9%, *NlPPP1-Y1* and *NlPPP1-Y2* is 89.5%, while *NlPPP1-Y* is divergent from other PPP1s which showed about 54% identity with *NlPPP1-Y1* and *NlPPP1-Y2* (Table 3). The best matched sequence for NlPPP1-Y is PP1 isoform alpha3 from *Drosophila navojoa*, with 72.7% identity and 46% coverage at nucleotide level, 56% identity and 100% coverage at amino acid level.

**Table 3:** Amino acid and nucleotide sequence identities between the protein phosphatase 1 catalytic subunits from *D. melanogaster* and *N. lugens*.

	Dm PP1α	Dm PP1β	Dm PP1-Y1	Dm PP1-Y2	Dm PP1-D5	Dm PP1-D6	Dm PPN58A	Dm PPY55A	Nl PPP1α	Nl PPP1β	Nl PPP1-Y	Nl PPP1-Y1	Nl PPP1-Y2
**Percent amino acid identity**													
DmPP1α		71.5	49.1	59.0	53.0	50.6	53.7	55.0	70.1	65.4	52.3	63.1	62.5
DmPP1β	80.2		47.7	57.4	49.7	49.9	50.8	56.2	65.5	70.3	50.4	62.1	62.1
DmPP1-Y1	54.7	54.4		52.4	46.5	48.1	48.0	46.6	49.0	45.4	44.2	46.2	46.6
DmPP1-Y2	71.0	68.5	59.6		50.6	47.7	51.8	49.0	59.3	58.2	47.5	54.3	54.8
DmPP1-D5	57.6	54.8	53.1	61.8		47.7	46.8	51.4	47.5	47.2	46.2	48.2	48.5
DmPP1-D6	54.0	51.8	52.8	55.4	51.0		49.4	49.2	46.1	47.2	45.1	48.9	48.8
DmPPN58A	56.3	56.0	55.3	58.0	50.5	51.4		48.7	51.3	49.2	48.8	50.4	49.8
DmPPY55A	58.4	57.8	51.1	55.7	53.7	49.2	50.2		52.6	52.1	50.3	48.1	48.6
NlPPP1α	89.3	79.9	55.3	70.4	57.4	57.4	56.9	59.4		66.9	48.1	61.5	61.3
NlPPP1β	81.0	86.5	53.8	67.2	55.5	55.5	54.2	58.1	82.2		47.9	60.3	58.8
NlPPP1-Y	55.5	53.3	50.3	56.1	50.6	50.6	50.5	52.7	55.0	54.0		54.4	54.0
NlPPP1-Y1	71.8	68.9	50.6	63.1	55.5	53.5	55.1	53.5	70.2	70.8	55.8		89.5
NlPPP1-Y2	72.8	69.9	50.6	63.5	54.5	53.8	55.1	53.5	71.2	71.8	55.8	97.8	

The upper half shows the identitics of nucleotide sequences, and the lower half those of the deduced amino acid sequences.

### Phylogenetic and sequence alignment of PPP1

**Figure 2:**
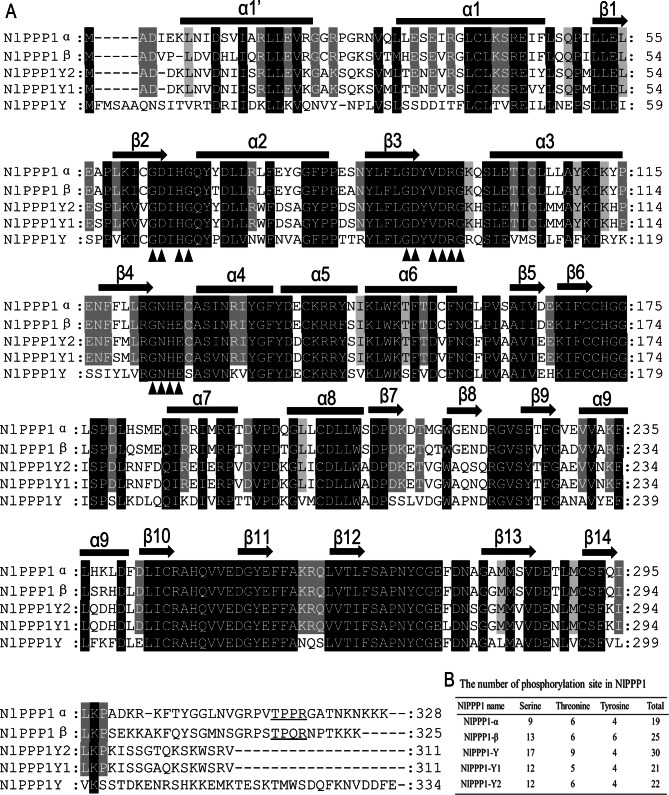
Amino acid sequences alignment of PPP1 from *N. lugens* using CLUSTAL X (**A**) and phosphorylation site analysis (**B**)*.* Identical residues are shown in black. Identical residues between NlPPPlα, NlPPPlβ, NlPPP1Y1 and NlPPP1Y2 but different from NlPPP1Y were marked with grey letters. Arrows blow the amino acids indicate the three signature motifs of PPP family. Cdk phosphorylation (TPP/QR) site were shown with underline. Secondary-structure elements were marked with arrows (β strands) and filled rectangles (α helices).

Blastp searches against the NCBI database revealed orthologues of NlPPP1 from other insects. The primary structures of the deduced amino acid sequences PPP1 from *N. lugens* were compared. Three signature motifs GDxHG, GDxVDRG, and GNHE (G, glycine; D, aspartic acid; x, any amino acid; H, histidine; V, valine; R, arginine; N, asparagine; E, glutamic acid) of PPP family within the catalytic domain were found. The conservative catalytic domain starting from the second α-helix (α1) and ending with the last β-strand (β14) as reported by Goldberg^32^ were showed in Fig. 2A. All PPP1s contain a Thr-Pro-Pro-Arg (TPPR) amino acid sequence segment at their carboxyl terminal, which is a consensus sequence for phosphorylation by cyclin-dependent kinases (Cdks) demonstrated in somatic cells^33–35^. In Drosophila and *N. lugens*, this TPPR segment is retained in DmPP1α (PP1α-96A), NlPPP1α, and NlPPP1β, but absent in DmPP1α1 (DmPP1α-87B), DmPP1α2 (DmPP1α-13C), and male-biased NlPPP1-Ys. As a characteristic of most Ser / Thr / Tyr protein kinases, a large number of potential phosphorylation site were also identified in NlPPP1, among them, there are 9 to 17 Ser sites, 5 to 9 Thr sites, and 4 to 6 Tyr sites (Fig. 2B). The number and composition of phosphorylation sites for each gene are also different. The minimum number of potential phosphorylation sites is 19 in NlPPP1-α and the maximum is 30 in NlPPP1-Y. At the nucleic acids level, the identities between *DmPP1α* and *NlPPP1α*, *NlPPP1-Y1*, *NlPPP1-Y2* and *NlPPP1-Y* are 70.1%, 63.1%, 62.5% and 52.3%, respectively. At the amino acid level, the identities are 89.3%, 71.8%, 72.8% and 55.5%, respectively. The identities of NlPPP1β with DmPPP1β are much higher both at the amino acid and nucleic acid level with 86.5% and 70.3% respectively (Table 3).

**Figure 3:**
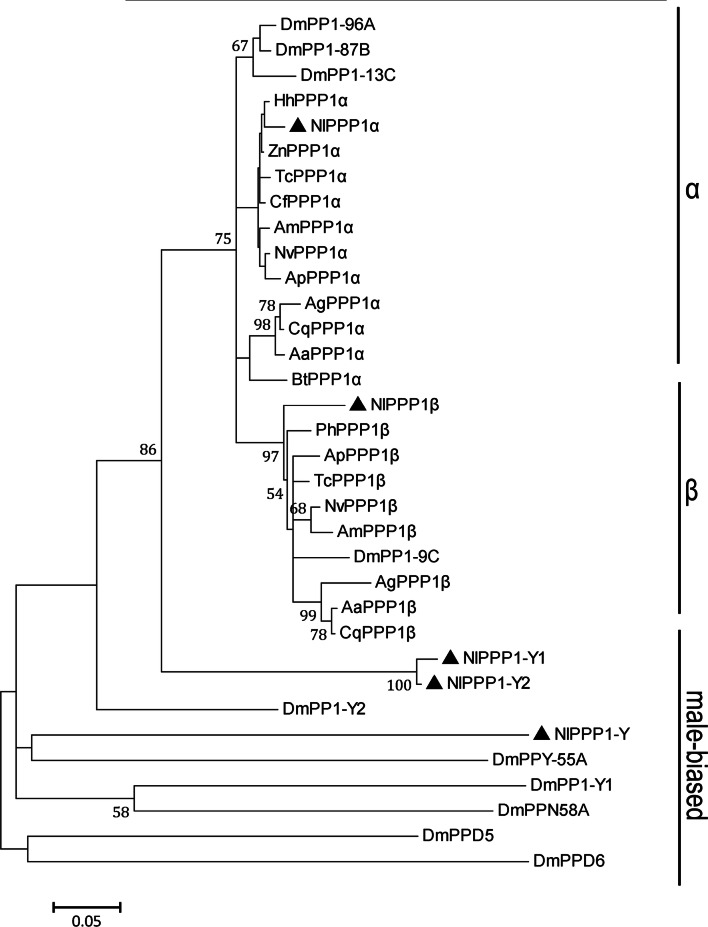
Phylogenetic analysis of PPP1 sequences from *N. lugens* and other insects. The phylogenetic tree of PPP1 homologs was constructed using the Maximum Likelihood method with MEGA5 software. Bootstrap values are shown in the nodes. Branch lengths are proportional to sequence divergence. The scale bar indicates the average number of amino acid substitutions per site. Sequences data were listed in Supplementary Table 1.

Phylogenetic tree was constructed using the MJ method to evaluate the molecular evolution relationships of the five PPP1s of *N. lugens* and other PPP1s from representative insect species. Five NlPPP1s were clustered into three classes (α, β and male-baised) (Fig. 3).

### Expression characteristic of *NlPPP1* in *N. lugens*

**Figure 4:**
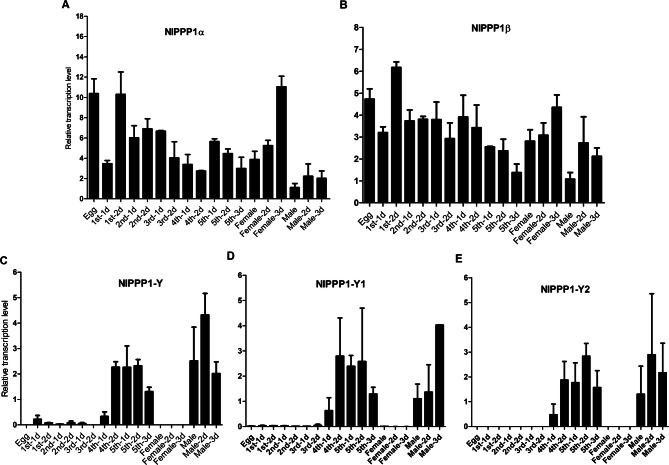
The developmental expression profile of five *NlPPP1s*. NlPPP1s expression value of males was converted to 1. Gene expression was normalized using β-actin and 18 s rDNA as reference gene.

The developmental expression profile of five *NlPPP1* in *N. lugens* was determined using RT-qPCR. *NlPPP1α* and *NlPPP1β* were expressed in all developmental stages and both sexes (Fig. 4A, B). In contrast, the transcription of *NlPPP1-Y, NlPPP1-Y1* and *NlPPP1-Y2* were restricted from 4th instar nymph to adult males whereas in females were nearly undetectable. The trace transcription of *NlPPP1-Y* and *NlPPP1-Y1* were observed from 1 to 3 instar nymph (Fig. 4C–E).

**Figure 5:**
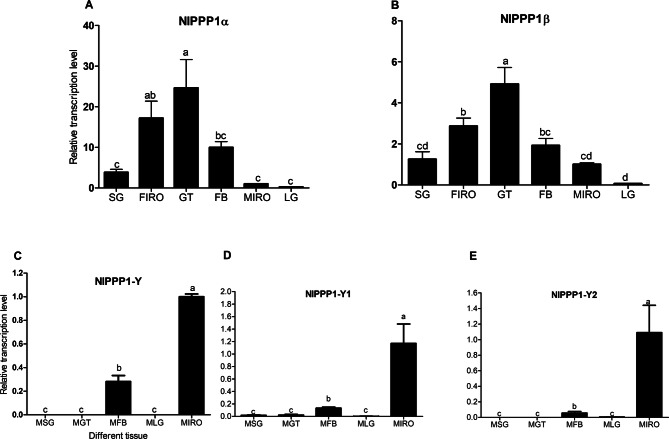
The expression pattern of *NlPPP1s* in various tissues from *N. lugens*. *NlPPP1s* expression value of MIRO was converted to 1. Duncan’s tests were used to determine differences between tissues. The histogram bars (mean ± SEM) labeled with the same letter are not significantly different at *p* < 0.05.

We then investigated the expression pattern in various tissues dissected from adults, including salivary glands(SG), fat bodies(FB), guts(GT), legs(LG), male internal reproductive organ(MIRO), and female internal reproductive organ (FIRO). The RT-qPCR results demonstrated that *NlPPP1α* and *NlPPP1β* showed significant higher expression in guts than in other tissues (Fig. 5A, B). To investigate the tissue-specific expression of male-biased *NlPPP1s*, total RNA was isolated from male tissues including SG, LG, GT, FB, and MIRO, for RT-qPCR analysis. The transcripts of *NlPPP1-Y*, *NlPPP1-Y1* and *NlPPP1-Y2* showed exclusive expression in MIRO, with relative expression levels in MIRO 13, 7, and 18 -fold higher than in the MFB respectively (Fig. 5C–E). Their transcripts were also detected in tissues SG, GT and LG at very low level. The trace expression may be caused by fat bodies contamination in dissection.

### dsRNA sequence analysis

Six dsRNAs targeted to different *NlPPP1* genes or different region were synthesized. The dsRNA name, length, targeted position and the largest length of 100% similarity stretch between the dsRNA and *NlPPP1s* were listed in Table 4. The specific dsRNAs were designed based on 3′-UTR of *NlPPP1β*, 5′-UTR of *NlPPP1-Y* and 5′-UTR of *NlPPP1-Y1* respectively. The sequence of dsNlPPP1α showed 100% identity with 34 bp stretch in *NlPPP1β*. dsNlPPP1-Y2 with 78 bp stretch in *NlPPP1-Y1*, dsNlPPP1Ys showed over 20 bp stretch in *NlPPP1-Y1* and *NlPPP1-Y2*. Moreover, dsNlPPP1Ys also showed over 50 bp stretch with more than 95% and 85% identity in *NlPPP1-Y1* and *NlPPP1-Y2* respectively.

**Table 4:** Details of the dsRNA designed in this study.

dsRNA name	Targeted gene	Position(site)	^a^NlPPP1α	^a^NlPPP1β	^a^NlPPP1-Y	^a^NlPPP1-Y1	^a^NlPPP1-Y2
dsNlPPP1α	NlPPP1α	ORF(345–1,140)	796	34	17	17	14
dsNlPPP1β	NlPPP1β	3-UTR(960–1,630)	7	669	5	7	6
dsNlPPP1-Y	NlPPP1-Y	5-UTR	6	7	533–704	8	7
dsNlPPP1-Y1	NlPPP1-Y1	5-UTR(1–524)	6	8	5	524	11
dsNlPPP1-Y2	NlPPP1-Y2	ORF(8–692)	11	7	11	78	685
dsNlPPP1Ys	NlPPP1-Y	ORF(114–890)	15	17	777	21	20

^a^The largest length (bp) of 100% similarity stretch between the dsRNA and genes.

### Influence of injection dsNlPPP1α and dsNlPPP1β on survival rate and fecundity

**Figure 6:**
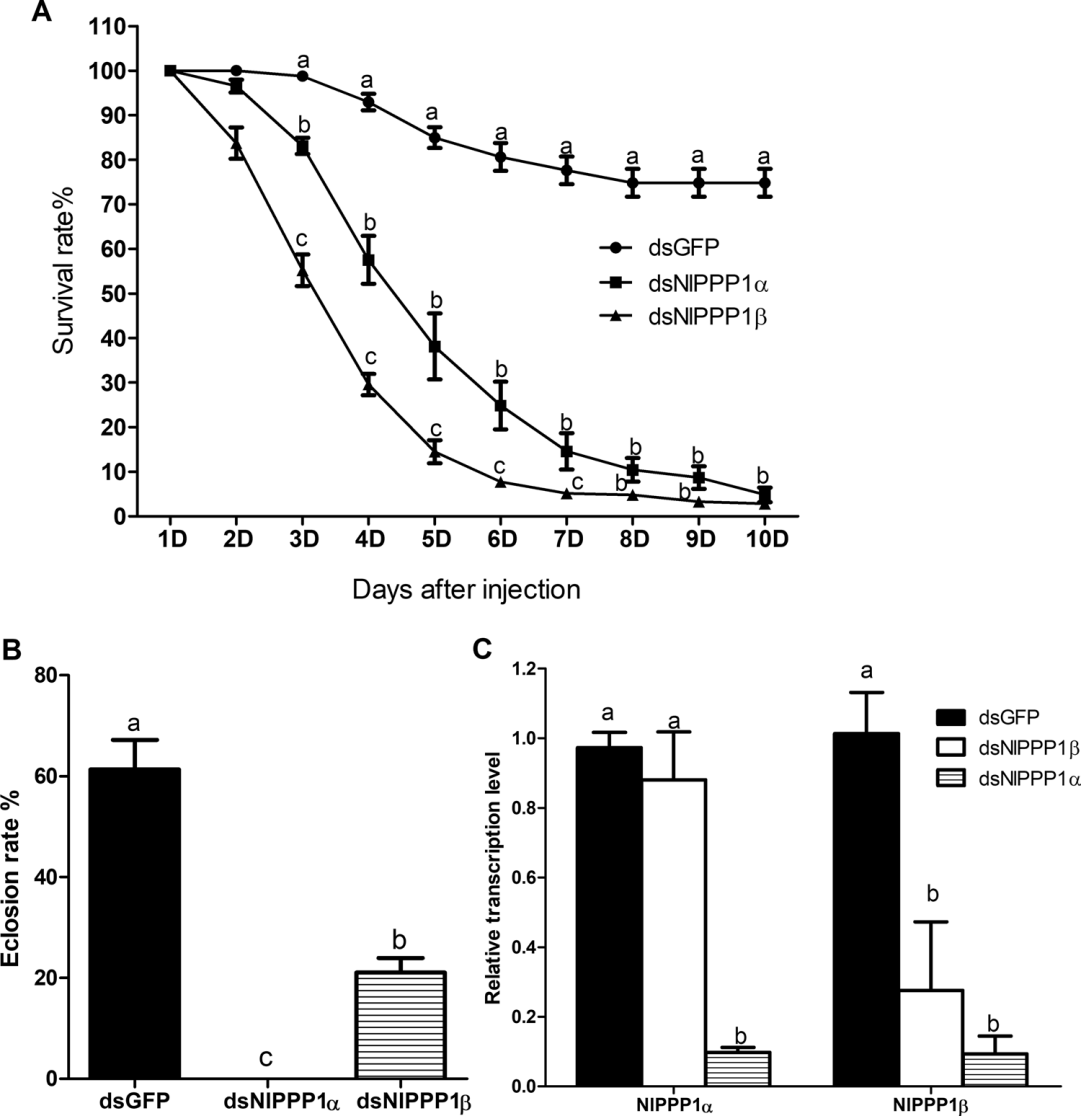
Effects of injection dsNlPPP1α and dsNlPPP1β on *N. lugens* survival rate (**A**), eclosion ratio (**B**) and inhibition of the transcription of two target genes (**C**). Gene transcription levels were relative to the control GFP normalized to the internal control (β-actin and 18 s rDNA). Duncan’s tests were used to determine differences between the treatment and control. The histogram bars (mean ± SEM, n = 5) with different letters are significant differences at *p* < 0.05.

Injection of dsNlPPP1α and dsNlPPP1β caused a significant decrease in the survivorship of *N. lugens*. The survival rate at 3rd day after injection was significantly lower in nymphs injected with dsNlPPP1α (83.1 ± 1.8%) and dsNlPPP1β (55.2 ± 3.5%) than with the dsGFP (100.0%). Ten days after injection, the survival rate of nymphs decreased to 4.8 ± 1.6% (dsNlPPP1α) and 2.8 ± 1.2% (dsNlPPP1β) (Fig. 6A). Almost no nymphs injected with dsNlPPP1α did reach to the adult stage. In the treatment of dsNlPPP1β, eclosion ratio was significantly reduced to 21.1 ± 5.8%, when compared to the dsGFP control (61.2 ± 13.2%) (Fig. 6B).

Fourth-instar nymphs were treated with dsRNA to assess the effects on reproduction. Because of high mortality, among 15 pairs, only 3 females injected with dsNlPPP1α and 4 females injected with dsNlPPP1β remained alive and available for oviposition. The mean number of eggs produced by dsNlPPP1α and dsNlPPP1β treated parent pairs was 196 and 133.3, respectively, significantly lower than that of control dsGFP (304 eggs/female).

*NlPPP1α* and *NlPPP1β* transcript levels in nymphs after the injection of dsNlPPP1α at 4th day were significantly down-regulated (Fig. 6C). dsNlPPP1α injection treatments led to reduced *NlPPP1α* and *NlPPP1β* expression approximately by 91.0% and 71.5% respectively, compared to the dsGFP control. The transcript levels of *NlPPP1β* decreased by 73.0% after the injection of dsNlPPP1β. However, no significant differences on transcript level of *NlPPP1*α in specimen injected dsNlPPP1β were observed.

### Influence of male-biased *NlPPP1* dsRNA injection on survival rate and fecundity

**Figure 7:**
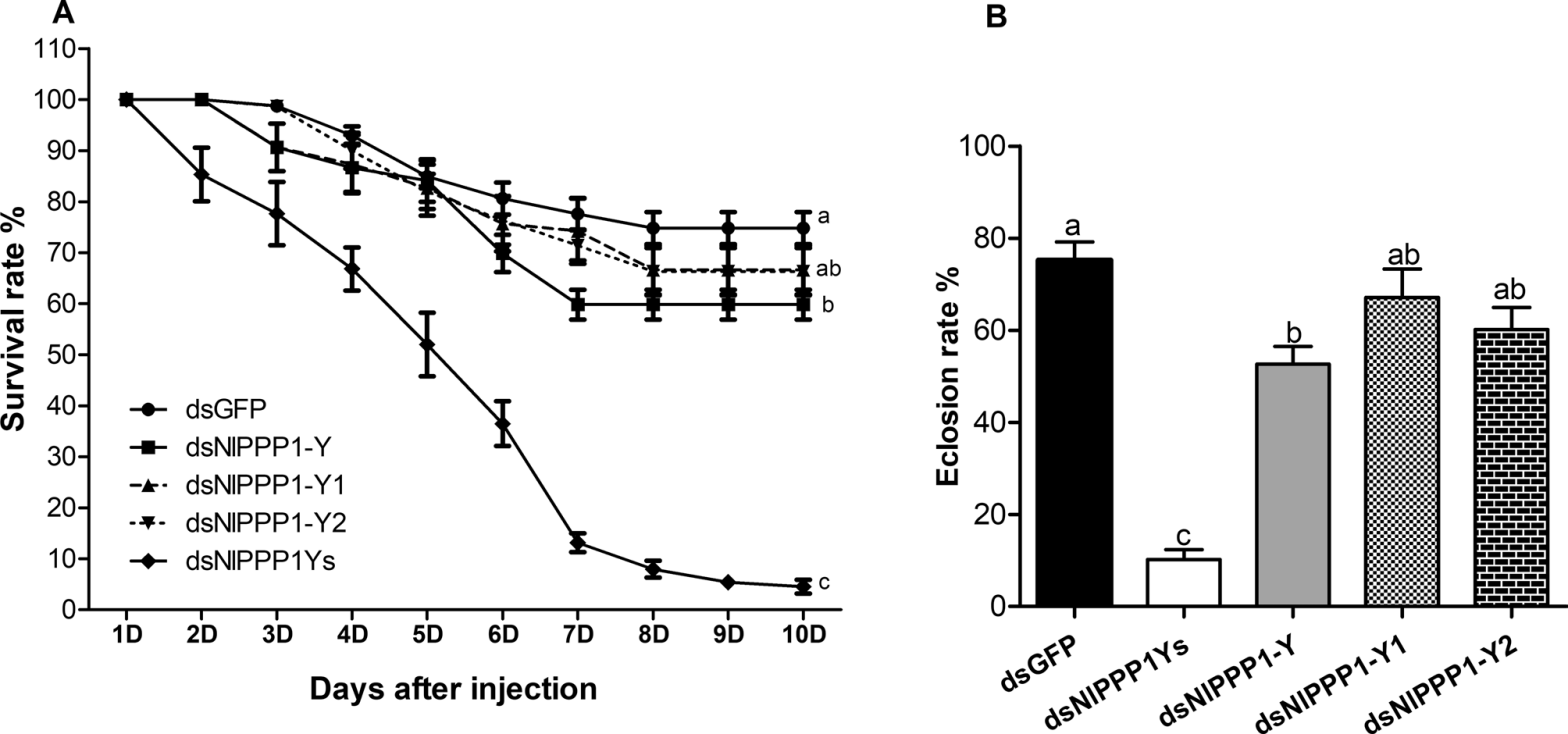
Effects of dsRNA injection of male-biased *NlPPP1* on *N. lugens* survival rate (**A**), Eclosion ratio (**B**). Gene transcription levels were relative to the control GFP normalized to the internal control (β-actin and 18 s rDNA). Duncan’s tests were used to determine differences between the treatment and control. The histogram bars (mean ± SEM) labeled with the same letter are not significantly different at *p* < 0.05.

The survival rate of nymphs injected with dsNlPPP1Ys began to decrease at 4th day after injection and decreased to 4.5 ± 3.0% at 10th day (Fig. 7A). Only slightly decrease was observed with dsNlPPP1-Y (59.8 ± 5.1%), dsNlPPP1-Y1 (66.6 ± 10.6%) and dsNlPPP1-Y2 (66.3 ± 10.4%) when compared with the dsGFP control (74.8 ± 7.0%). Eclosion ratio was significantly reduced in nymphs injected with dsNlPPP1Ys (10.2 ± 5.0%) and dsNlPPP1-Y (52.6 ± 8.5%). No significant reduction was observed in the treatment injected with dsNlPPP1-Y1 (67.1 ± 13.8%) and dsNlPPP1-Y2 (60.2 ± 10.7%) when compared to the dsGFP control (75.2 ± 8.8%) (Fig. 7B).

**Figure 8:**
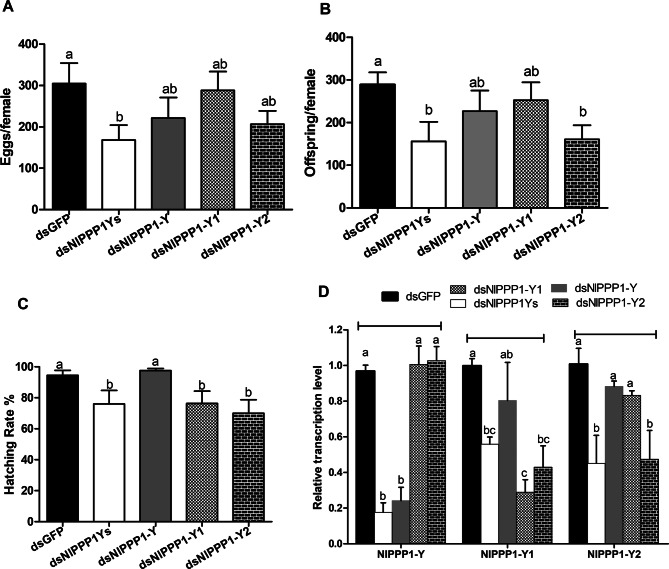
Effects of dsRNA injection of male-biased *NlPPP1* on *N. lugens* eggs amount (**A**), offspring (**B**), hactching rate (**C**) and gene transcript levels (**D**). Fourth-instar nymphs (3-day old) *N. lugens* were used for dsRNA injection. The histogram bars in A, B and C show mean value (n = 15 independent biological replicates), the histogram bars in D show mean relative gene expression (n = four independent biological replicates).The error bars represent standard error of mean. Gene transcription levels were relative to the control GFP normalized to the internal control (β-actin and 18s rDNA). Duncan’s tests were used to determine differences between the treatment and control. The histogram bars labeled with the same letter are not significantly different at *p* < 0.05.

The dsNlPPP1Ys and dsNlPPP1-Y2-treated males mated with the control females led to significantly reduced oviposition amount by 44.7% (from 304 to 168 eggs/female) and by 31.9% (from 304 to 207 eggs/female), and the offspring significantly decreased by 55.4% (from 289 to 129) and 44.3% (from 289 to 161), relative to dsGFP-treated males mated with the control females respectively (Fig. 8A, B). The dsNlPPP1-Y-treated males mated with the control females, however, showed slightly reduced oviposition amount and offspring, and no effect on hatchability. When the control females mated with dsNlPPP1Ys, dsNlPPP1Y1, or dsNlPPP1Y2 treated males, their hatching rate significantly reduced. The hatchability decreased by 19.6% (from 94.5% to 76.0%), 19.3% (from 94.5% to 76.3%) and 26.0% (from 94.5% to 69.9%), respectively, relative to dsGFP-treated males mated with the control females (Fig. 8C). RNAi efficiency by dsRNA injection was confirmed by RT–qPCR. The transcript levels of *NlPPP1-Y, NlPPP1-Y1* and *NlPPP1-Y2* at the male internal reproductive organ decreased by 82.3 ± 0.1%, 44.1 ± 0.1% and 54.8 ± 0.2% respectively after dsNlPPP1Ys injection, indicating that the transcript of *NlPPP1-Y* had been effectively silenced. The transcript levels of *NlPPP1-Y1* and *NlPPP1-Y2* decreased by 57.1 ± 0.2% and 52.5 ± 0.3% respectively after the injection of dsNlPPP1-Y2, *NlPPP1-Y1* decreased by 71.1 ± 0.1% after the injection of dsNlPPP1-Y1 and *NlPPP1-Y* decreased by 75.8 ± 0.1% after injection of dsNlPPP1-Y when compared with the control dsGFP (Fig. 8D). RT–qPCR result showed that the transcript levels of male-biased *NlPPP1* genes, *NlPPP1-Y*, *NlPPP1-Y1* and *NlPPP1-Y2,* were significantly reduced in males injected with dsNlPPP1Ys, and injection with dsNlPPP1-Y2 not only led to reduced expression of the target gene *NlPPP1-Y2* but also the reduced expression of *NlPPP1-Y1.*

### dsNlPPP1Ys treated males showed malformed internal reproductive organ

**Figure 9:**
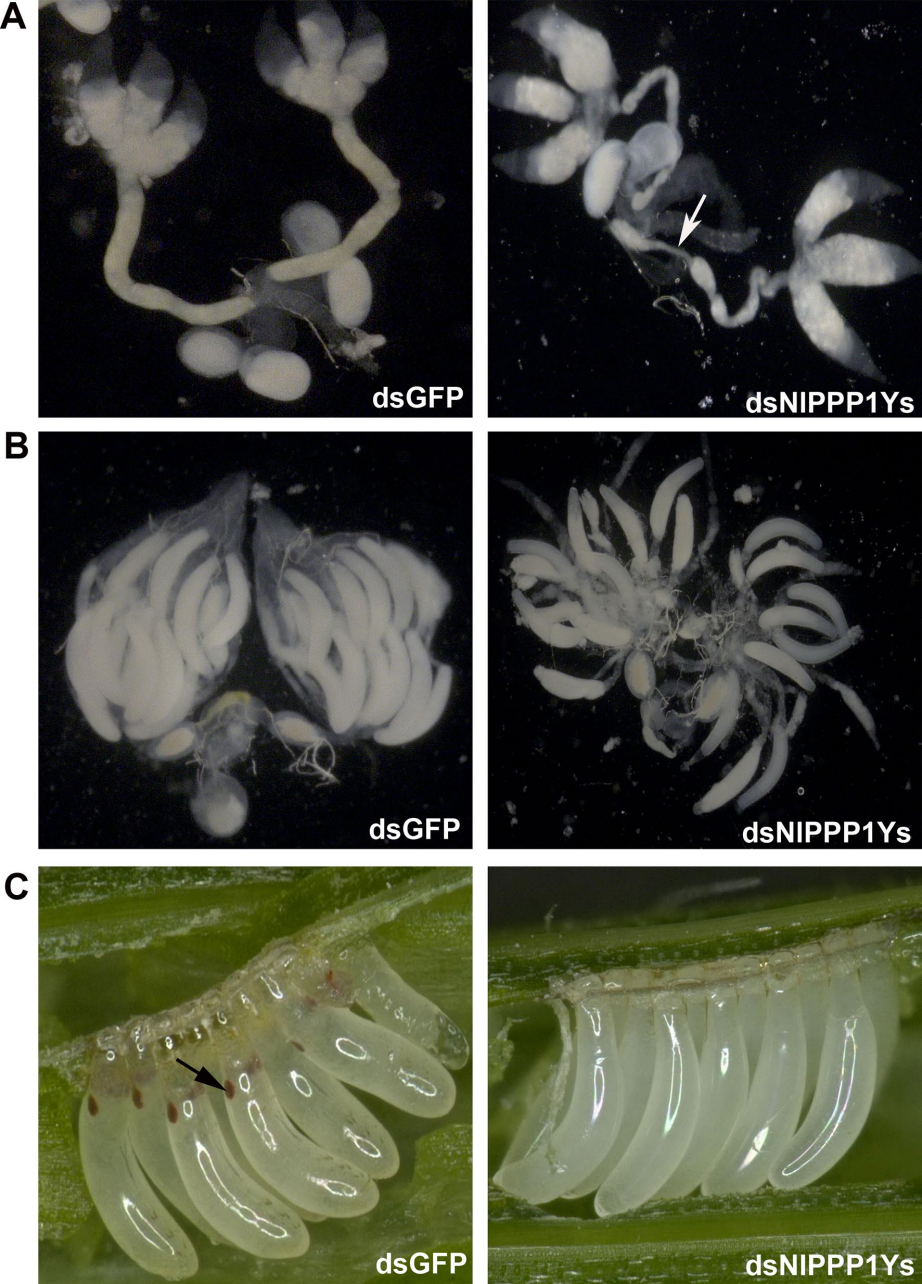
The influence of dsNlPPP1Ys treated males on morphology of IRO of males (**A**) and their mating partners (**B**) and eggs (**C**). Fourth-instar nymphs (3-day old) *N. lugens* were used for dsRNA injection. IROs were dissected from the male 2 days after emergence and from the female 4 days after emergence and photographed. n > 10. (**A**) The thinned vas deferens observed in dsNlPPP1Ys-treated males was marked with arrow. (**B**) Fewer ripe banana-shaped oocytes in females mated with dsNlPPP1Ys-treated males were observed. (**C**) Unfertilized eggs without red eyespot in dsNlPPP1Ys-treated group. The red eyespot of fertilized eggs in dsGFP-treated control was marked with black arrow.

The internal reproductive organ prepared from males dsNlPPP1Ys-♂, dsNlPPP1-Y1-♂, dsNlPPP1Y2-♂ and dsNlPPP1-Y-♂ after 2 days emergency, were dissected and photographed. Significant malformation of vas deferens was observed in males from treatment dsNlPPP1Ys-♂, compared to dsGFP-treated males, dsNlPPP1-Ys treatment led to thining vas deferens (Fig. 9A). No clear morphology difference was observed in dsNlPPP1-Y1-♂, dsNlPPP1-Y2-♂ and dsNlPPP1-Y-♂ treatment. The internal reproductive organ prepared from females (control-♀) mated with experimental males (dsNlPPP1Ys-♂), was also dissected and photographed. The ovarioles contained fewer ripe banana-shaped oocytes compared to controls at 4 day after emergency (Fig. 9B). Microscopic observation showed that the eggs scraped from the leaf sheaths were unfertilized in dsNlPPP1Ys, dsNlPPP1-Y1, or dsNlPPP1-Y2-treated group (Fig. 9C). dsNlPPP1Ys treatments led to decreased total protein in internal reproductive organ from males and their partners at 2 days after emergency (Table 5).

**Table 5:** Mating with *dsNlPPP1Ys treated* males led to reduced protein content in IRO.

Treatments	ug/MIRO	ug/FIRO
Male × female		
dsGFP × control	14.83 ± 0.56a	63.24 ± 3.25a
dsNlPPP1Ys × control	12.75 ± 0.38b	51.18 ± 1.25b

The data in the table are means ± SEM (N = 4). A total of 40 male internal reproductive organs (4 replicates, 10 individuals in each replicate) and 20 female internal reproductive organs (4 replicates, 5 individuals in each replicate) were dissected from males or females 2 days after emergence, respectively. Different letters indicate significant difference between dsGFP treated control group and dsNlPPP1Ys treated group at p < 0.05.

## Discussion

One of the most widespread mechanisms of post-translational regulation of proteins is the addition of phosphate by protein kinase, this phosphorylation is antagonized by protein phosphatases. The antagonistic actions of protein kinases and protein phosphatases are of equal importance in determining the degree of phosphorylation of each substrate protein^8^. Five different isforms of PPP1 defined by three signature motifs GDxHG, GDxVDRG, and GNHE within the conserved 30 kDa catalytic domain were identified from *N. lugens*. The constitutively expressed *NlPPP1α* and *NlPPP1β* were highly conserved which has higher to 86% identity with *DmPPP1α* and *DmPPP1β* respectively at the amino acid level. Down-regulation of *NlPPP1α* and *NlPPP1β* transcription resulted in 90% mortality in ten days and no nymph emergence. The dsNlPPP1α sequence from ORF region of *NlPPP1α* has 34 bp stretch of 100% identity with *NlPPP1β*, which contribute to the transcription reduction of *NlPPP1β* in nymphs injected with dsNlPPP1α. Only down-regulation the *NlPPP1β* with dsNlPPP1β sequence from 3′-UTR region of *NlPPP1β* resulted in 90% mortality in ten days and 80% reduction of eclosion ratio. Our results showed that silencing *NlPPP1α* and *NlPPP1β* are semilethal, therefore they are essential genes in *N. lugens.*

The male-biased expressed *NlPPP1Y, NlPPP1-Y1* and *NlPPP1-Y2* were more divergent than non-sex biased PPP1 gene. Increasing evidences suggest that genes related to sex and reproduction change much faster between species than those limited to survival^36^. As demonstrated in Drosophila, male-biased genes evolve faster than unbiased genes in both coding sequence and expression level^37–40^. Specific silencing either *NlPPP1-Y1* or *NlPPP1-Y2* gene resulted in no or only slight mortality. Specific silencing of *NlPPP1-Y* with dsNlPPP1-Y resulted in 40% mortality, whereas silencing of 3rd instar nymphs using dsNlPPP1Ys designed against ORF of *NlPPP1-Y* resulted in 90% mortality and 80% reduction of eclosion rate, in which three male-biased *NlPPP1-Y*, *NlPPP1-Y1* and *NlPPP1-Y2* were silenced. This result suggests that this group of phosphatases has overlapping function allowing the compensation for the lack of one or the other member of the gene family. In *D. melanogaster*^41^*, Heliothis virescens*^42^ and *N. lugens*^43^, male accessory gland proteins transferred to adult females via mating can regulate egg maturation and stimulate oogenesis, ovulation and oviposition. Our result showed that females mated with *NlPPP1-Y*, *NlPPP1-Y1* and *NlPPP1-Y2* silenced males led to a reduction in eggs amount and hatchability, whereas mated with *NlPPP1-Y1* or *NlPPP1-Y2* silenced males only led to a reduction in hatchability. Silenced *NlPPP1-Y* had no significant effect on both the oviposition amount and hatching rate. This result suggested that *NlPPP1-Y1* and *NlPPP1-Y2* may play more important roles in spermatogenesis and fertilization, and *NlPPP1-Y* mainly involved in development of male *N. lugens*. The physiological role of the male-specific phosphatases is still elusive, although the location and timing of their transcription as well as the conservation of their male-biased expression hint a specific role in reproduction. So the fine function of *NlPPP1* in male development, sperm development and fertility needs to be clarified.

RNAi silences gene expression through the production of small interfering RNAs (siRNAs). In *Caenorhabditis elegans*, the pairs having high degree of sequence similarity with the RNAi clones (100% over 25 bp, ≥ 94% over 50 bp, ≥ 89% over 100 bp, ≥ 84% over 200 bp and ≥ 81% over 300 bp) were predicted to exhibit off-target cross-reaction^44^. The question of over how much length and how much similarity is necessary to observe off-target cross-reaction remains open in *N. lugens*. In our study, all target NlPPP1 genes were successfully silenced by using dsRNAs containing over 25 bp with 100% similarity. The efficient RNAi effect was also observed between *NlPPP1-Y1* with dsNlPPP1Ys and *NlPPP1-Y*2 with dsNlPPP1Ys. *NlPPP1-Y1* has 21 bp with 100% similarity or 54 bp with 95% similarity with the dsNlPPP1Ys. *NlPPP1-Y2* has 20 bp with 100% similarity or 59 bp with 85% similarity with the dsNlPPP1Ys. RT-qPCR showed the expression of *NlPPP1-Y1* and *NlPPP1-Y2* reduced by 44.1% and 54.8% respectively in males injected with dsNlPPP1Ys when compared with males injected with dsGFP.

The overuse of conventional synthetic insecticides caused not only the serious detrimental effect on the environment but also the emergence of pest insect resistance to insecticides^2^. RNAi-based pest control strategies are emerging as environment friendly and species-specific alternatives for the use of conventional pesticides^45^. As the critical molecular switch in cell, protein phosphatase is a preferably considered target when designing RNAi-based pest control strategy as it affects numerous proteins dephosphorylation. *NlPPP1-Y*, *NlPPP1-Y1*, and *NlPPP1-Y2* have low homology to known PPP1. The dsNlPPP1Ys sequence presents 100% similarity stretch with other organism is short than 10 bp. This means that the sequence of dsNlPPP1Ys varies greatly among insect species and the possibility of off-target effects is tiny. The dsNlPPP1Ys also showed ability to inducing high mortality rate, low eclosion rate and fecundity by interfering three male-biased *NlPPP1* genes. In the application of RNAi to conserved genes at the cDNA level, the 3′-UTR is a good candidate sequence^46^. The dsNlPPP1β designed based on 3′-UTR also showed ability to inducing high mortality rate, low eclosion ratio and fecundity by silencing *NlPPP1β* genes. Therefore, *NlPPP1-Ys* would be a high efficient potential target gene used for *N. lugens* control. The selected dsNlPPP1β and dsNlPPP1Ys can be the preferred target sequence used for *N. lugens* control by means of RNAi.

## Supplementary information

Supplementary Table 1
