# Supplementary material for: Identification and functional analysis of five genes that encode distinct isoforms of protein phosphatase 1 in Nilaparvata lugens
Source: Sci Rep. 2020 Jul 2;10:10885. doi: 10.1038/s41598-020-67735-7 (PMC7331678; doi:10.1038/s41598-020-67735-7)
Supplement: Supplementary file 1 — Supplementary Table 1 [file 41598_2020_67735_MOESM1_ESM.docx]

**Identification and functional analysis of five genes that encode distinct isoforms of protein phosphatase 1 in *Nilaparvata lugens***

Weixia Wang^a^, Tingheng Zhu^b*^, Fengxiang Lai^a^, Pinjun Wan^a^, Qi Wei^a^ and Qiang Fu^a*^

Table S1. Protein sequences sources used in Fig 3.

| Sequence name | Accession NO. | Species name |
| --- | --- | --- |
| CqPPP1β | XP_001843526 | *Cluex quinquefasciatus* |
| AaPPP1β | XP_001663366.1 | *Aedes aegypti* |
| AgPPP1β | XP_312797 | *Anopheles gamibae* |
| AmPPP1β | XP_623273 | *Apis mellifera* |
| NvPPP1β | XP_001604472.1 | *Nasonia vitripennis* |
| TcPPP1β | XP_966417.2 | *Tribolium castaneum* |
| ApPPP1β | XP_001944422 | *Acythosiphon pisum* |
| PhPPP1β | EEB19394 | *Pediculus humanus corporis* |
| CqPPP1α | XP_001849462 | *Cluex quinquefasciatus* |
| AaPPP1α | XP_001653770 | *Aedes aegypti* |
| AgPPP1α | XP_309483 | *Anopheles gamibae* |
| ApPPP1α | XP_001945902.2 | *Acythosiphon pisum* |
| NvPPP1α | XP_001602738 | *Nasonia vitripennis* |
| AmPPP1α | XM_392943.3 | *Apis mellifera* |
| TcPPP1α | XP_001813974 | *Tribolium castaneum* |
| CfPPP1α | XP_014213558.1 | *Copidosoma floridanum* |
| ZnPPP1α | XP_021913699.1 | *Zootermopsis nevadensis* |
| HhPPP1α | XP_014274729.1 | *Halyomorpha halys* |
| BtPPP1α | XP_018912512.1 | *Bemisia tabaci* |
| DmPP1-9C | NP_727418.1 | *Drosophila melanogaster* |
| DmPP1-13c | NP_524921.1 | *Drosophila melanogaster* |
| DmPP1-87B | NP_524937.1 | *Drosophila melanogaster* |
| DmPP1-96A | NP_001262919.1 | *Drosophila melanogaster* |
| DmPP1-Y2 | 窗体顶端  NP_001015497.4窗体底端 | *Drosophila melanogaster* |
| DmPP1-Y1 | 窗体顶端  NP_001104152.3窗体底端 | *Drosophila melanogaster* |
| DmPPN58A | 窗体顶端  NP_477384.1窗体底端 | *Drosophila melanogaster* |
| DmPPD6 | 窗体顶端  NP_524947.1窗体底端 | *Drosophila melanogaster* |
| DmPPY55A | 窗体顶端  NP_001286554.1窗体底端 | *Drosophila melanogaster* |
| DmPPD5 | NP_524707.1 | *Drosophila melanogaster* |
